# Individualized time windows enhance TMS-EEG signal characterization and improve assessment of cortical function in schizophrenia

**DOI:** 10.1007/s00406-024-01859-z

**Published:** 2024-07-06

**Authors:** Gema Mijancos-Martínez, Alejandro Bachiller, Inés Fernández-Linsenbarth, Sergio Romero, Leidy Y. Serna, Vicente Molina, Miguel Ángel Mañanas

**Affiliations:** 1 Biomedical Engineering Research Centre (CREB), Department of Automatic Control (ESAII), Universitat Politècnica de Catalunya - BarcelonaTech (UPC), Barcelona, Spain; 2 Institute of Research Sant Joan de Déu, Barcelona, Spain; 3 Psychiatry Department, School of Medicine, University of Valladolid, Valladolid, Spain; 4 CIBER of Bioengineering, Biomaterials and Nanomedicine (CIBER-BBN), Madrid, Spain; 5 Psychiatry Service, Clinical Hospital of Valladolid, Valladolid, Spain; 6 Neurosciences Institute of Castilla y Léon (INCYL), University of Salamanca, Salamanca, Spain

**Keywords:** TMS-EEG, Characterization, Individualization, TEP, Time window, Schizophrenia

## Abstract

Transcranial magnetic stimulation and electroencephalography (TMS-EEG) recordings are crucial to directly assess cortical excitability and inhibition in a non-invasive and task-free manner. TMS-EEG signals are characterized by TMS-evoked potentials (TEPs), which are employed to evaluate cortical function. Nonetheless, different time windows (TW) have been used to compute them over the years. Moreover, these TWs tend to be the same for all participants omitting the intersubject variability. Therefore, the objective of this study is to assess the effect of using different TWs to compute the TEPs, moving from a common fixed TW to more adaptive individualized TWs. Twenty-nine healthy (HC) controls and twenty schizophrenia patients (SCZ) underwent single-pulse (SP) TMS-EEG protocol. Firstly, only the HC were considered to evaluate the TEPs for three different TWs in terms of amplitude and topographical distribution. Secondly, the SCZ patients were included to determine which TW is better to characterize the brain alterations of SCZ. The results indicate that a more individualized TW provides a better characterization of the SP TMS-EEG signals, although all of them show the same tendency. Regarding the comparison between groups, the individualized TW is the one that provides a better differentiation between populations. They also provide further support to the possible imbalance of cortical excitability/inhibition in the SCZ population due to its reduced activity in the N45 TEP and greater amplitude values in the N100. Results also suggest that the SCZ brain has a baseline hyperactive state since the TEPs of the SCZ appear earlier than those of the HC.

## Introduction

Transcranial Magnetic Stimulation (TMS) is a novel, non-invasive technique that alters the cortical neuronal excitability through a magnetic pulse [1]. It allows a better understanding of the cortical circuitry [1, 2]. TMS stimulation triggers characteristics event related potentials known as TMS-evoked potentials (TEPs) that can be recorded simultaneously using electroencephalography (EEG). In the EEG signals, TEPs are characterized by changes in voltage across time spreading through the scalp. They are originated by the post-synaptic potentials of the pyramidal neurons and interneurons stimulated by the TMS-pulse; thus, they are time-locked to the TMS-pulse [3]. TEPs are defined regarding their polarity (negative or positive; N or P) and their latency (in ms, time after TMS-pulse onset) [4]. The evaluation of the TMS-EEG signals, focusing on TEP amplitude and its spatial distribution, has been used by researchers to study different brain disorders, such as schizophrenia, bipolar and mood disorders or substance use disorders. In this context, most studies have traditionally targeted the motor cortex although there is an increasing trend in research that involves stimulating the dorsolateral prefrontal cortex (DLPFC) [3].

Schizophrenia is a chronic psychotic brain disorder affecting 24 million people worldwide, according to the World Health Organization [5]. This disorder is characterized by structural and functional alterations in the brain, manifesting in symptoms such as hallucinations, delusions and disordered speech or thoughts. Patients often present blunt affect and atypical behaviors, including social withdrawal [6, 7]. Therefore, researches have used TMS-EEG data to investigate this complex disorder. Studies involving schizophrenic (SCZ) population have revealed an alteration in the excitation-inhibition balance [3, 8]. Specifically, research suggests alterations in cortical inhibition mechanisms, thought to be mediated by the GABAergic neurons in the DLPFC [3, 9]. Neuropathological and in vivo evidences support that a cortical excitatory/inhibitory disequilibrium plays a role in the pathophysiology of this disorder [10, 11]. Concretely, the most consistently replicable TEPs in current research, arising from a single TMS-pulse at the DLFPC or motor cortex (M1) stimulation, include P30, N45, P60, N100 and P180 [3, 4, 12, 13]. Among these, the N100 deflection is probably the most reproducible of the TMS-evoked potentials components [14, 15]. Since the N100 amplitude of the TMS-evoked potential has been reported to index the glutamate/GABA balance in vivo [16], it is necessary to improve the methods for comparing this TEP between patients and controls to increase our understanding of the underpinnings of psychotic disorders.

Despite an extensive list of characterized TEP reported in schizophrenia literature, there has been a consistent variability in the computation of TEP amplitude and topography due to the use of heterogeneous time windows (TW). A TW is defined by the latency and the window length considered to compute the desired parameters used for signal characterization. While some researchers have used a fixed TW, employing a time-constant, common window for all participants, either based on data from the study or not [12, 17–20], others have employed a more individualized TW centered around the manually selected TMS-peak for each participant [21–23]. The diverse procedures for creating the TWs, which will define the TEPs, introduce a variability in signal characterization, thereby influencing the final TEP analysis. Moreover, in cases of fixed TW or lack of subject individualization, certain TEPs might be missed and excluded from the analysis.

For these reasons, we aimed to analyze the topographical differences resulting from the utilization of different TWs and their impact when comparing two different populations (HC and SCZ patients). Furthermore, we hypothesized that a better TMS-EEG signal characterization is achieved when personalized latencies are used rather than using a generalized common TW. In addition, we believe that a personalized TW has the potential to more precisely target the desired TEP, consequently facilitating the identification of more significant differences between populations.

In the current study, we employed single-pulse (SP) TMS-EEG signals from the HC group to assess various TW reported in the literature. This involved characterizing the TEPs in terms of amplitude, latency and spatial distribution to determine if the different TWs yielded distinct information. Lastly, the TEPs of SCZ patients and HC were evaluated regarding their amplitude, topography and latencies for the different TWs in order to determine which TW better reflect the differences between the two groups.

## Materials and methods

### Participants

Twenty-nine right-handed healthy controls (HC; 13 males, 27 ± 12 years) and twenty schizophrenia patients (SCZ; 11 males, 35 ± 13 years) participated in the study. The SCZ group was composed by 11 first episodes and 9 chronic patients. Patients had been diagnosed by a psychiatrist according to the criteria of the *Diagnostic and Statistical Manual of Mental Disorders 5th edition*, considering their current mental state, clinical records, and information from relatives. Participants meeting any of the following exclusion criteria were excluded: (a) intelligence quotient under 70; (b) history of present or past substance dependence (excluding caffeine and nicotine); (c) head trauma with loss of consciousness; (d) neurological or mental diagnosis besides schizophrenia (for patients); (e) any current neurological or psychiatric diagnosis (controls); (f) receiving additional treatment affecting central nervous system; (g) potentially at risk for undergoing TMS. Prior the inclusion in the study, participants provided informed written consent after full written information. The study was endorsed by the local ethics committee and adhered to the ethical standards in the Helsinki Declaration of 1975, as revised in 2008.

### Transcranial magnetic stimulation

Seventy-five monophasic single TMS pulses were administered to the DLPFC of each participant using a figure-of-8 coil (MCF-B70) and a MagProX100 stimulator (MagVenture, Denmark). The administration of the pulses was semi-randomized, occurring between 5 and 7 s to prevent anticipation of the next pulse. The specific stimulation site was the midpoint of a line between the F3 and F5 electrodes, with a 45º rotation relative to the midline. This positioning was chosen for its accurate estimation of the left DLPFC and low inter-subject variability in the absence of neuronavigational equipment, [24, 25]. Participants were seated comfortably, looking directly ahead with their eyes open during the pulse administration. The intensity of the pulses was set to 120% of the Resting Motor Threshold (RMT), determined over the motor cortical region following the relative frequency method [26].

### EEG recording

Alongside with DLPFC stimulation, EEG was acquired using a 64-channel system amplifier (Brain Vision [Brain Products GmbH]) following the 10–10 international system at a sampling frequency of 25 kHz. Sixty-one channels were used for recording brain signals whereas the remaining 3 were allocated for vertical and horizontal electrooculography and electromyography. The impedance for each electrode was kept below 5 kΩ, and channels were referenced over Cz during acquisition.

### TMS-EEG pre-processing and processing

The pre-processing and processing of TMS-EEG data were performed using MATLAB (R2021b; The MathWorks Inc., Natick, MA, USA) and FieldTrip [27].

First, the TMS-EEG data was segmented into 2-seconds epochs centered around the TMS-pulse onset. Due to the irretrievable nature of the samples of the TMS-pulse, within each epoch, samples of the pulse and around it (from − 1ms to 10ms) were removed and cubic interpolated [28]. Then, the data was re-referenced to common average. Secondly, independent component analysis (ICA) was applied to reduce artefacts. Independent components were manually and blindly selected by three different experts, based on time-frequency maps, trial-averaged amplitude, spatial distribution and activation maps [28–30]. Thirdly, bad channels interpolation and bad trial rejection were automatically performed. Finally, a baseline correction was applied using an interval of 800 ms before the TMS-pulse onset, and the data was downsampled to 5 kHz and band-pass filtered between 0.5 Hz and 70 Hz.

Artifact-free data was trial averaged for each subject to obtain TMS-evoked potentials (TEPs). It is noteworthy that only signals from HC were used for TW analysis, whereas data from both populations was considered for the characterization of the TMS-EEG signal.

### Time window (TW) analysis

Several TW previously employed in different studies were analyzed [17, 18, 29, 31, 32]. The aim was to compare these TWs concerning the topography of the HC population to understand their impact on the characterization of the TMS-EEG signals. The variables used to describe the TW were the width or the window’s length, and the latency, as the time instant around which the TW is centered. Both variables are measured in milliseconds (ms). Depending on the combination of these variables, different TW can be defined:

#### Fixed TW

Four different TW are defined according to the literature depending on the specific TEP under study: N45 (35-60ms), P70 (60-80ms), N100 (85-140ms) and P180 (150-230ms) [17, 18, 31].

#### GMA TW

Grand-mean average (GMA) TW were based on the grand-mean average signal of the HC population. The GMA signal was computed by first averaging all trials for each subject and then performing an average for all subjects. Subsequently, the channel displaying the most prominent projection of the TEP in the GMA signal was identified, and the onset time of the TEP was used as the center of the TW (latency). Finally, TWs of 10 ms width around the latency were used for early TEPs (N45 and P60), while 30 ms width TWs were created for late TEPs (N100, P140, and P180) [29, 32].

The same procedure was replicated for the SCZ population, considering the GMA signal of the SCZ signals.

#### Personalized TW

The third type of TW assessed was the Personalized TW. For each subject, the latency of each TEP was determined on the same channel obtained in the GMA TW method. Similarly, the onset of the TEP for each subject was used as the center of the subject’s TW and the width TW was adjusted analogously to the GMA TW. Therefore, for the Personalized TW, each subject has a different TW latency. Nonetheless, the width remains the same for all participants, corresponding to the width of the GMA TW. A summary of the 3 TW evaluated is shown in Table 1.

**Table 1:** Range, latency and width of the different type of time windows for each TEP. Note that for the fixed TW there is no P140 TEP

TEP	N45	P60	N100	P140	P180
**Fixed TW**					
Latency (ms)	47.5	70	112.5	-	190
Width (ms)	20	20	55	-	80
Range (ms)	35–60	60–80	85–140	150–230	
**GMA TW**					
Latency (ms)	43.6	63.4	118.4	136.8	214.8
Width (ms)	10	10	30	30	30
**Personalized TW**					
Latency (ms)^a^	43.8 ± 2.9	64.2 ± 9.4	115.9 ± 11	149.7 ± 15.8	208 ± 25.2
Width (ms)	10	10	30	30	30

^a^In the Personalized TW, each subject has their own latency for each TEP. The latency shown in the table are the mean and the standard deviation

### Signal characterization

To characterize the response to the TMS-pulse, the topographical distribution and the mean amplitude of the TEPs were computed for each TW. In addition, for the Personalized TW, the latency of the TEPs was determined. Consequently, the response to the stimulus was evaluated based on the magnitude of the TEPs, their distribution throughout the brain and their time of appearance.

### Statistical analysis

To analyze the TW and assess whether differences existed between them, a statistical topography study was conducted using the paired sample Wilcoxon singed-rank test. Similarly, a Wilcoxon rank sum test was used to perform a statistical topography study between conditions (HC and SCZ patients). The same test was performed for TEP amplitude and latency.

## Results

### TW comparison

**Fig. 1:**
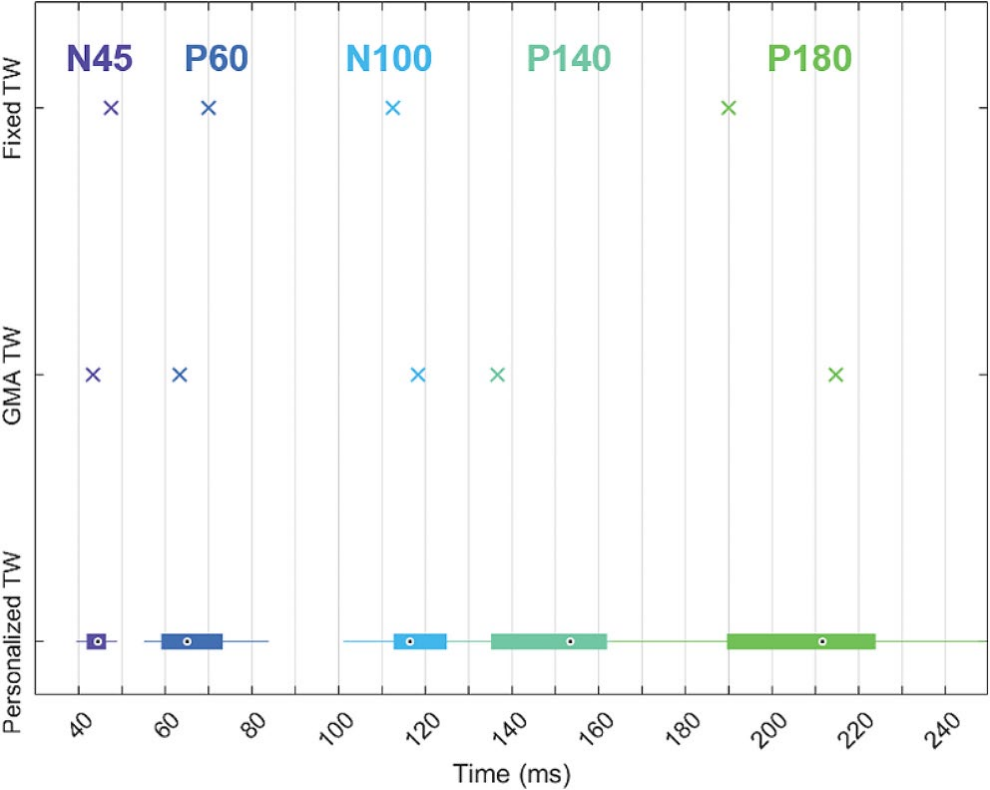
Latencies for each TEP and type of TW (from top to bottom: Fixed TW, GMA TW and Personalized TW)

The distribution of latencies for HC for each TEP is shown in Fig. 1. GMA latencies of the early TEPs (N45 and P60) are shorter than the fixed latencies proposed in the literature, whereas the late TEPs (N100 and P180) exhibit higher latencies. The GMA latency values consistently fall within the interquartile range of 25–75% of the Personalized TW latencies, whereas the latency of the Fixed TW is outside of this range in the N45 and P140. As for the latter TEP, it is not commonly reported in the literature, hence, there is no fixed latency for this TEP in the state-of-the art.

**Fig. 2:**
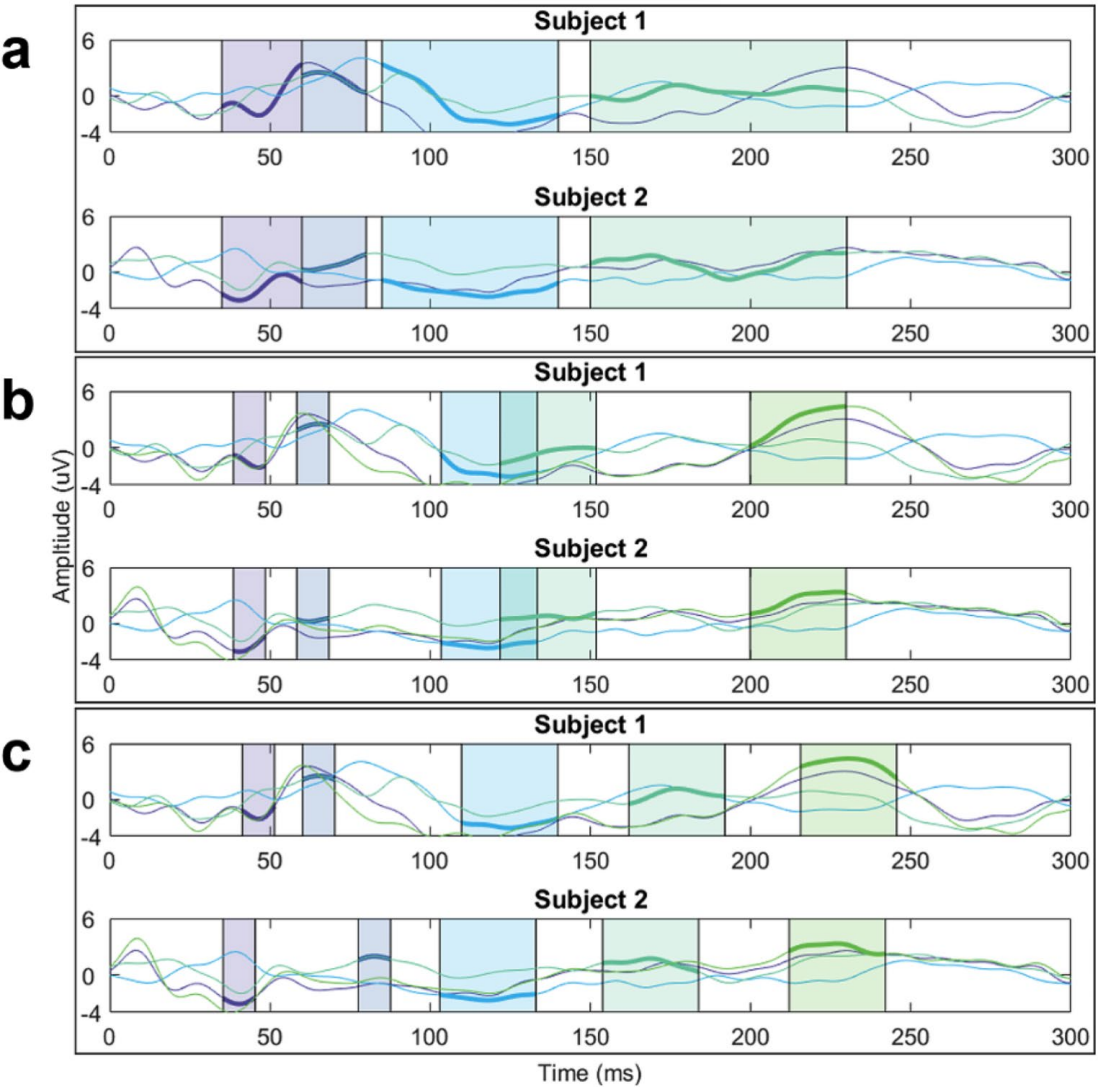
Time windows marked for 2 controls. For each TEP, the channel obtained in the GMA method is depicted. As the Fixed TW does not evaluate the P140 TEP, its representation has one channel less. (**a**) Fixed TW, (**b**) GMA TW and (**c**) Personalized TW

An example of the different TW applied to 2 subjects is depicted in Fig. 2. As observed, using data-dependent TWs, such as the GMA or the Personalized TW, allows for narrowing the width of the TW and being more precise by centering the TW to each peak.

**Fig. 3:**
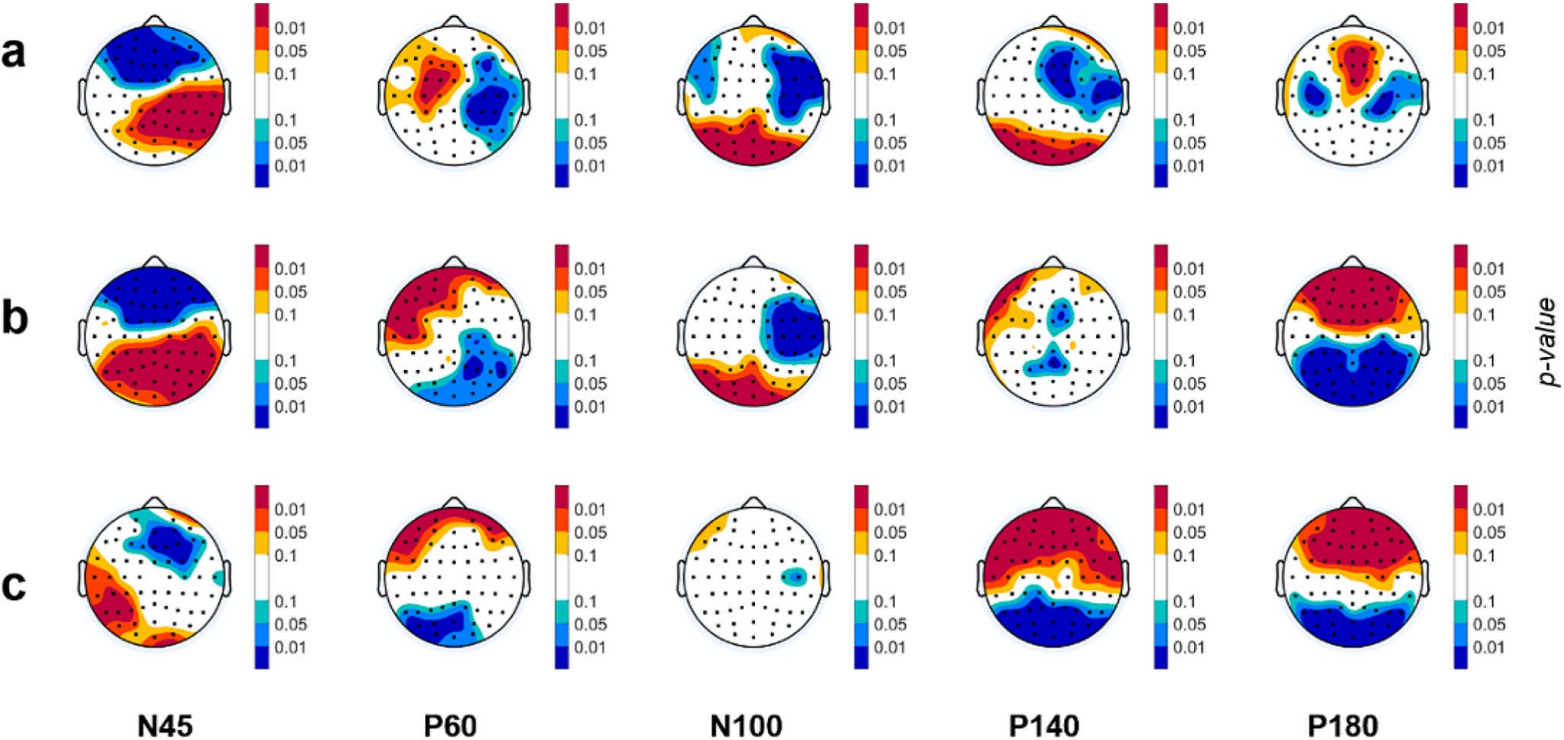
Statistical topography of the comparison of the TW for each TEP. (**a**) GMA vs. Fixed TWs; (**b**) Personalized vs. Fixed TWs and (**c**) Personalized TW vs. GMA TW. Red colors indicate that the amplitude on the first TW is higher than the second group and blue color the opposite. Light colors denote tendency (p-values between 0.1 and 0.05), medium intensity colors indicate p-value between 0.05 and 0.01 and dark colors refer to p-values below 0.01

Remarkable differences emerged among the three different topographical distribution of TW (Fig. 3). The first row (Fig. 3a) illustrates the statistical differences between the GMA and Fixed TWs. These TWs maintain consistency across all subjects, but they use different widths and latencies. Statistical differences are identified for all TEPs. In particular, the N45 wave presents more statistically significant electrodes than the other TEPs, whereas P180 shows the fewest differences. The N45 wave exhibits alterations in amplitude across nearly the entire cerebral cortex. Changes for P60 reach statistical significance in central electrodes, whereas for N100 and P140, differences are observed in the occipital and right parietal areas. Finally, the electrodes that account for most of the observed variance in P180 are located in the frontal and central regions.

Regarding the Personalized TW, it exhibits more electrodes with statistically significant differences compared to the Fixed TW (Fig. 3b). N45 and P180 present the largest differences, with: almost all electrodes yielding p-values < 0.01. Differences in P60 are evident in frontal and occipital electrodes. Differences in N100 are observed in occipital and right parietal regions. The P140 wave shows statistically significant differences in fewer electrodes located at central and frontal regions. On the other hand, when comparing the Personalized TW with the GMA TW (Fig. 3c), there are fewer differences in the early TEPs. Statistically significant differences in N100 are observable only between frontal and occipital electrodes. Contrarily, size effects are more prominent for late TEPs.

### Signal characterization

#### Topographic distribution of the TEPs

**Fig. 4:**
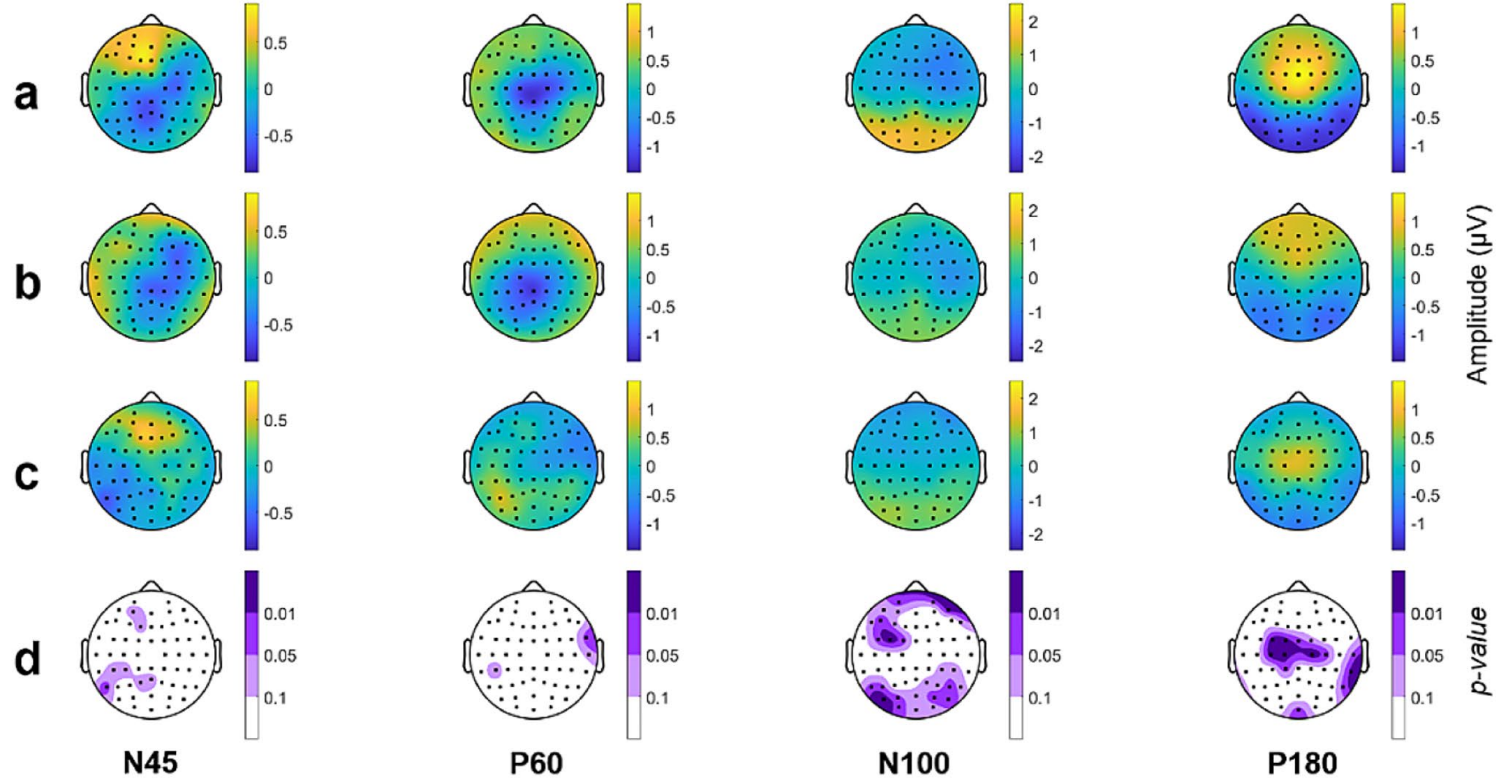
Topography of each TEP for the Fixed TW. (**a**) SCZ patients, (**b**) HC population, (**c**) difference (SCZ-HC) and (**d**) statistical topography SCZ vs. HC. In **a**), **b**) and **c**) the topoplots exhibit the amplitude across the cerebral cortex. In **d**) the p-value of each electrode of the comparison SCZ vs. HC is depicted, those marked with light purple indicated tendency (0.05 < p-value < 0.1), medium purple indicates statistical significance with a p-value between 0.01 and 0.05 and dark purple indicates p-value < 0.01

**Fig. 5:**
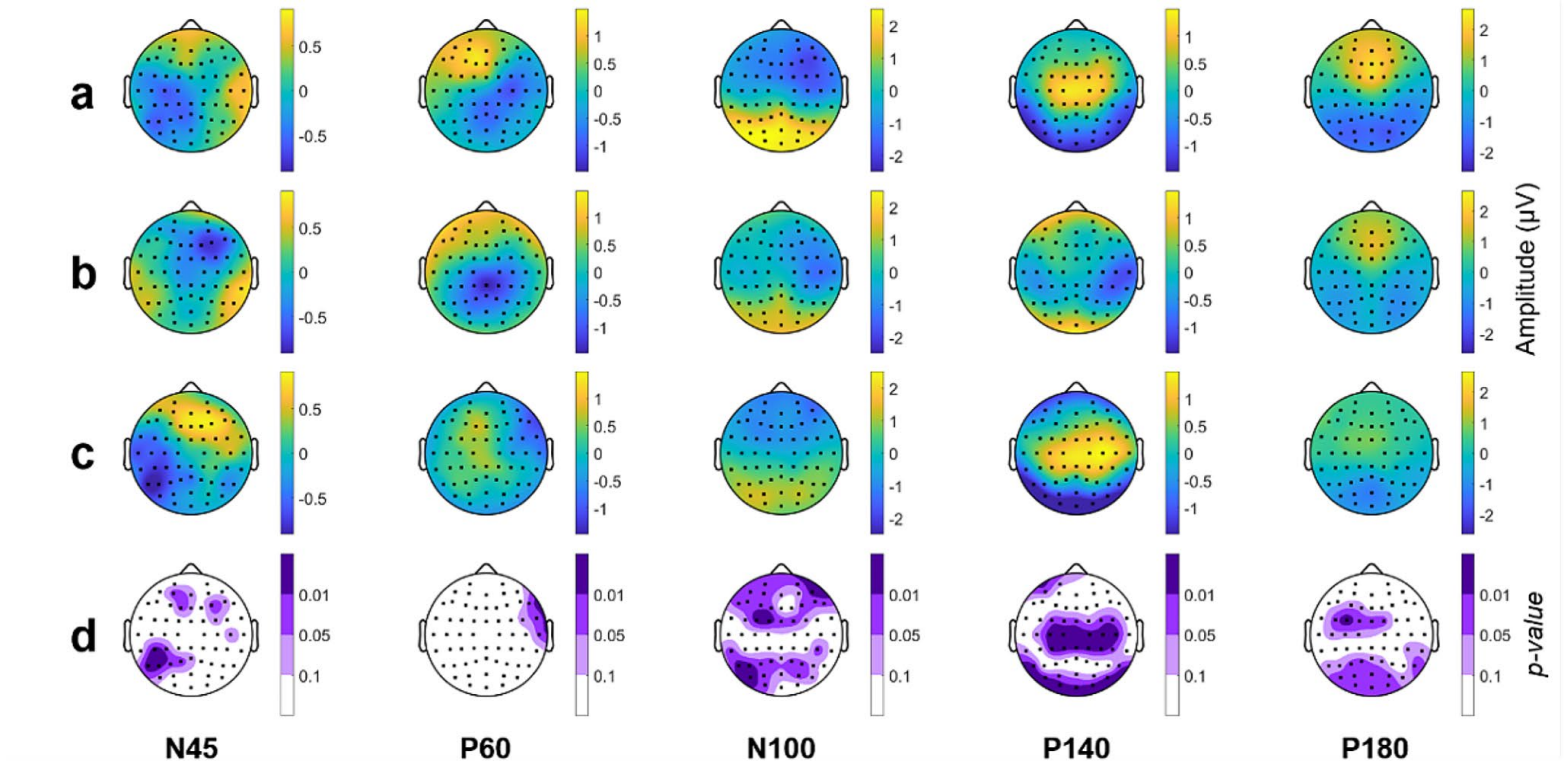
Topography of each TEP for the GMA TW. (**a**) SCZ patients, (**b**) HC population, (**c**) difference (SCZ-HC) and (**d**) statistical topography SCZ vs. HC. In **a**), **b**) and **c**) the topoplots exhibit the amplitude across the cerebral cortex. In **d**) the p-value of each electrode of the comparison SCZ vs. HC is depicted, those marked with light purple indicated tendency (0.05 < p-value < 0.1), medium purple indicates statistical significance with a p-value between 0.01 and 0.05 and dark purple indicates p-value < 0.01

**Fig. 6:**
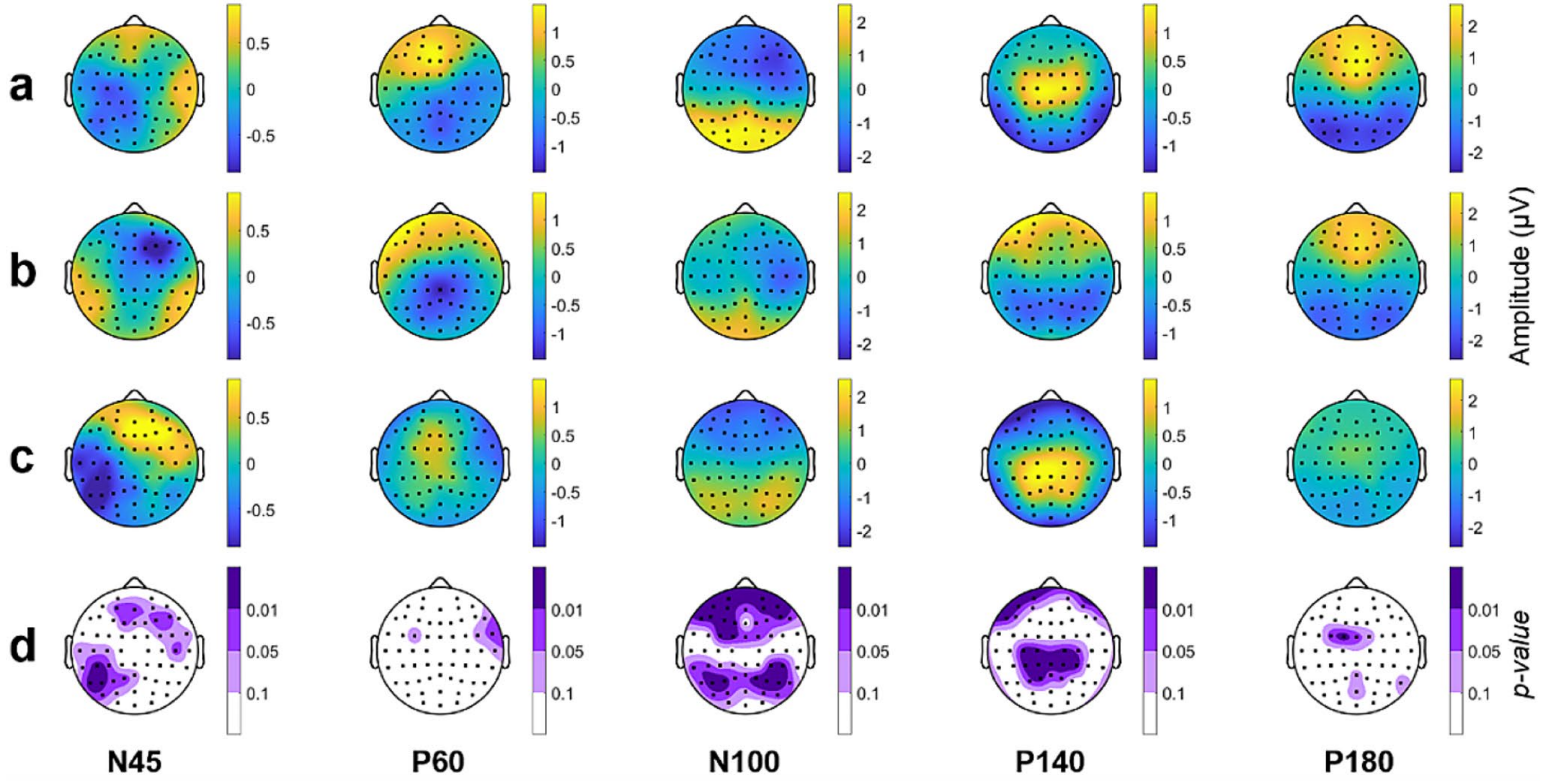
Topography of each TEP for the Personalized TW. (**a**) SCZ patients, (**b**) HC population, (**c**) difference (SCZ-HC) and (**d**) statistical topography SCZ vs. HC. In **a**), **b**) and **c**) the topoplots exhibit the amplitude across the cerebral cortex. In **d**) the p-value of each electrode of the comparison SCZ vs. HC is depicted, those marked with light purple indicated tendency (0.05 < p-value < 0.1), medium purple indicates statistical significance with a p-value between 0.01 and 0.05 and dark purple indicates p-value < 0.01

Figures 4, 5 and 6 represent the topographical distribution for each TEP for both populations (SCZ and HC), the topographic difference between populations, and the statistically significant differences between populations for each TW.

The spatial distribution of HC and SCZ using a Fixed TW closely resembles each other for some TEPs (Fig. 4). Differences are observable in N45, N100, and P180. In SCZ patients, N45 amplitude is higher in the left-frontal electrodes, although it does not reach statistical significance. In the case of N100, the SCZ group exhibits a greater voltage level in parietal electrodes and a lower level in the frontal region, both reaching statistical significance when compared to the HC population. Finally, the P180 wave of SCZ patients displays an amplitude increase, particularly in central electrodes, and these differences also reach statistical significance.

Similarly, Fig. 5 illustrates the topographical distribution for the GMA TW. The spatial distribution of N45 differs between the two groups, with a more negative pattern (lower amplitude) in the right frontal area for the HC population, although only a few electrodes have p-values lower than 0.05. No significant differences are observed between HC and SCZ groups in P60. Nevertheless, differences in amplitude values become more pronounced for late TEPs. N100 presents statistically significant differences in almost all brain regions except some central electrodes, with the SCZ group having higher amplitude values in the parietal area and lower values in the frontal area compared to the healthy group. For the potentials P140 and P180 TEPs the differences in amplitude are primarily in the central and parietal regions, with larger disparities in the central area where the SCZ group has greater amplitude values than the HC population.

Figure 6 illustrates the spatial distribution of the TEPs for both populations, along with their differences and statistical topography, specifically focusing on the Personalized TW. In this case, SCZ patients exhibit higher values of amplitude for N45 in the right-frontal brain area and lower amplitude in the left-occipital region. Regarding N100, statistically significant differences are observed in frontal and centro-parietal electrodes, with a greater amplitude in the frontal electrodes for SCZ patients and a smaller one in the centro-parietal brain area. In the case of P140, the topography of SCZ group exhibits a higher amplitude in the central electrodes compared to the HC group, reaching statistically significance (*p* < 0.01). Finally, there are fewer electrodes with statistically significant differences between populations regarding the P180 potential.

#### TEP amplitude and latency

**Fig. 7:**
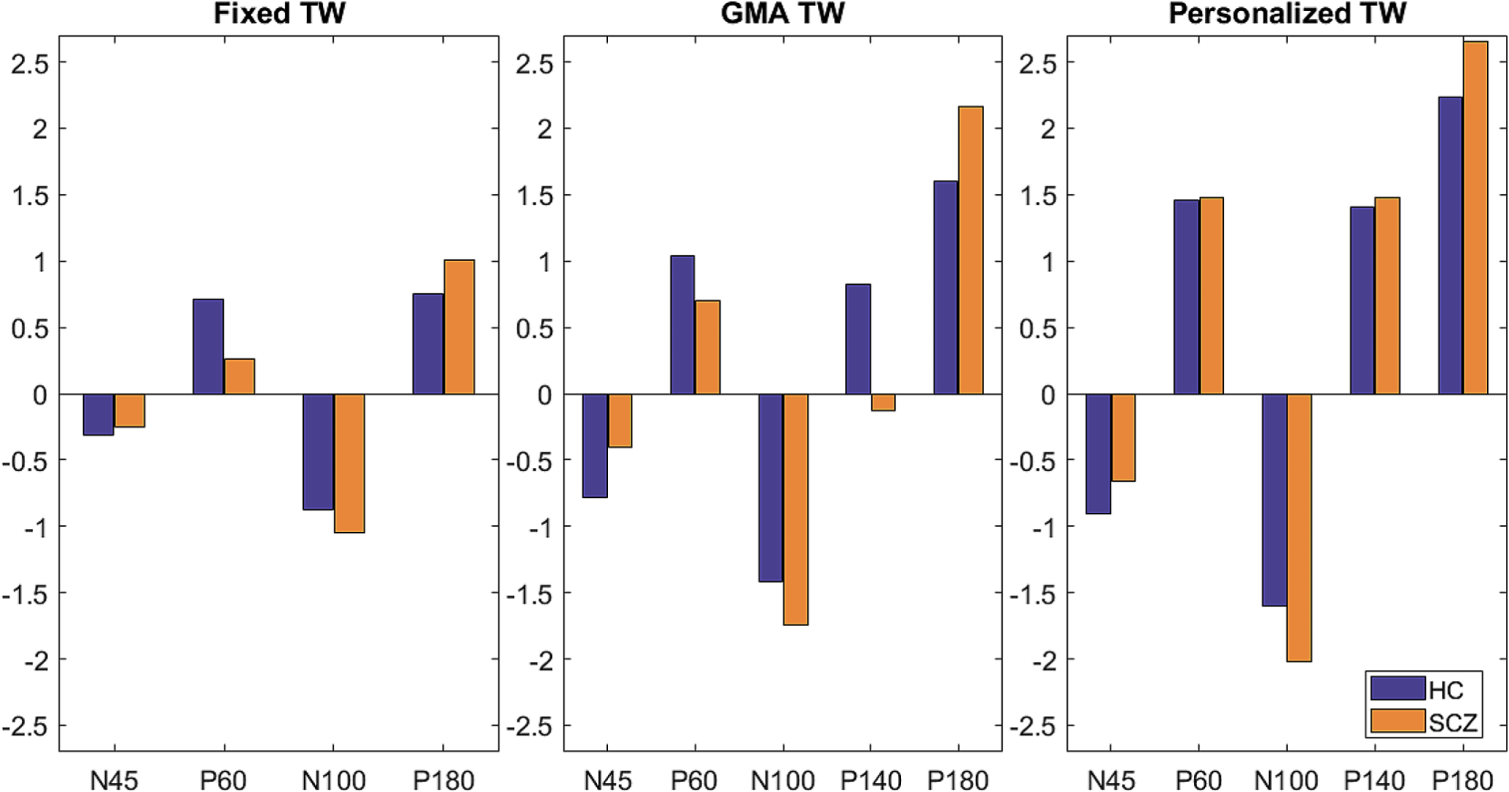
TEP amplitudes for the three types of TW in the channel obtained in the GMA TW methodology (left to right: Fixed, GMA and Personalized TWs) and both population groups (HC purple, SCZ orange)

As observed in Fig. 7, amplitudes values are greater when the TEPs are evaluated in a more individualized TW. For the GMA TW, the P140 wave for SCZ patients exhibits negative values, and no significant differences between populations are identified.

When examining the mean amplitude of the TEPs of the HC group across the different TW, no statistically significant differences are observed between Fixed and GMA TWs, nor between GMA and Personalized TWs. Nonetheless, the comparison of the amplitudes between Fixed TW and Personalized TW, the P60, N100, and P180 potentials exhibit p-values below 0.01, and the p-values for the remaining TEPs range between 0.05 and 0.08.

Concerning the SCZ population, there is statistical significance in the amplitude of the P140 when comparing Fixed and GMA TWs (p-value = 0.002) and GMA and Personalized TWs (p-value = 0.0001). Furthermore, P180 also exhibits statistically significant differences in amplitude when evaluating Fixed and GMA TWs (p-value = 0.01). Similar to the healthy population, the p-values for P60, N100, and P140 are non-significant.

**Fig. 8:**
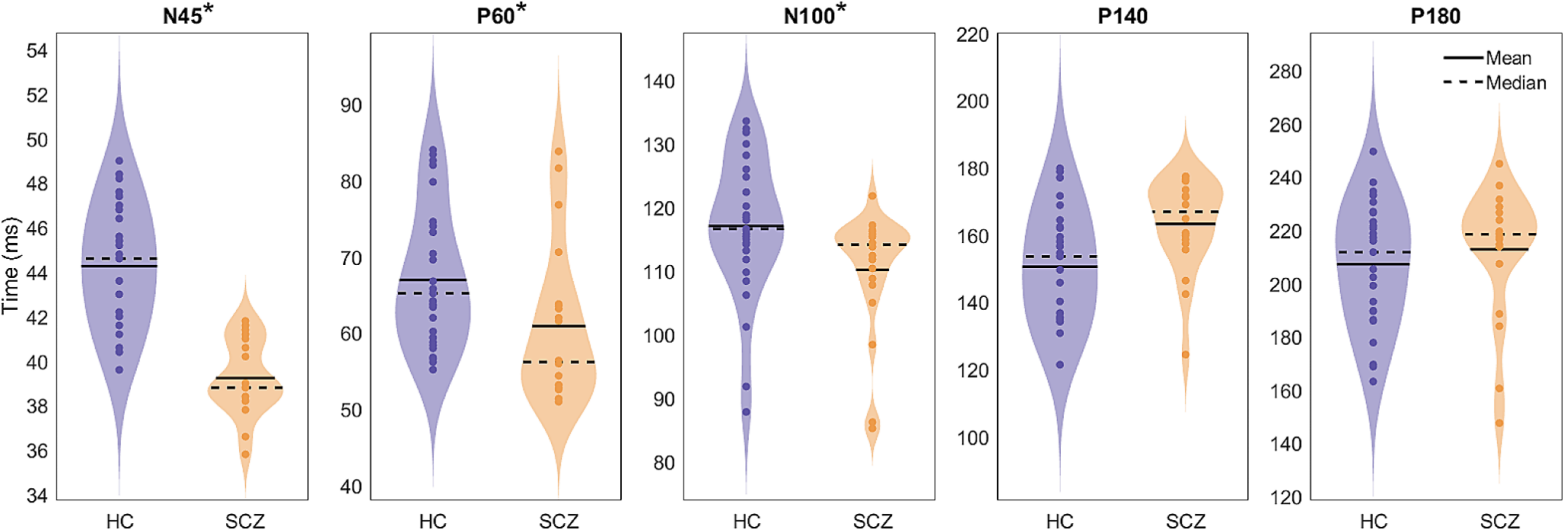
Violin plots of the latencies of HC (purple) and SCZ (orange) for each TEP in the Personalized TW. The TEP with the asterisk have a statistically significant p-value (*p* < 0.05) when evaluated between populations

The latencies of each TEP for both groups are represented in the violin plots of Fig. 8. For early TEPs and N100, the onset of the potentials occurs earlier in the SCZ population than in the healthy group, with p-values lower than 0.01 for N45 and P60, and less than 0.05 for N100. On the other hand, the onset of the late TEPs occurs earlier in the HC than in the SCZ (p-value < 0.01 for P140).

In summary, the TW that exhibits fewer differences in the distribution of the TEPs between populations is the Fixed TW. Both, the GMA and Personalized TWs display similar scalp voltage distribution. N100 and P140 potentials show more pronounced differences across populations for all three TW. Concerning amplitudes, their values increase with the personalization of the TW. Lastly, early TEPs appear earlier in the SCZ population than in the HC population, whereas the opposite occurs for late TEPs.

## Discussion

The outcomes of this study underscore the significant influence of both the latency and the width of the time window employed characterizing the TMS-evoked potentials, as these factors wield a substantial impact on the final results. Moreover, the use of an individualized TW for each subject enhances the characterization of the single-pulse TMS-EEG signal and maximizes the discernible differences between populations. Although the most pronounced differences are observed for an individualized TW, in all three TW exhibit consistent trends in differences among populations, particularly in the same brain regions. Therefore, a non-data dependent TW may offer a comprehensive overview of ongoing events, while data dependent TWs (i.e. GMA and Personalized TWs) provide more precise topographical localization.

The observed differences between SCZ and HC populations offer additional support for an imbalance in cortical excitability/inhibition within the SCZ group. Furthermore, the findings suggest that the schizophrenic brain may exhibit a baseline hyperactive state, as indicated by the earlier onset of early TEPs in the SCZ population.

### TW comparison for HC population

The choice of different TW significantly influences the characterization of the single-pulse TMS-EEG, leading to differences in parameters defining the TEP, such as amplitude and scalp distribution. As shown in Fig. 2, TWs with fixed latencies for all subjects (Fixed and GMA TW) either missed TEPs in some subjects or do not center the TEP within the window, resulting in an inaccurate evaluation of the potential. This is attributed to the inherent variability in the latencies of the TEPs, as it is illustrated in Fig. 1. While the latencies of N45, P60, N100, and P180 potentials using the GMA TW closely align with the median latency of the same TEPs when individually evaluated, there are instances where some TEPs are missed. The P140 potential, being less pronounced and distinct, is more prone to smoothing during averaging, leading to a poor match between the GMA latency and the median latency of the population. In contrast, the Fixed TW is often centered distantly from the median of the personalized latencies, missing even more TEPs for some subjects. Nevertheless, due to the large width of the Fixed TW, this decentralization of the TEP can sometimes be mitigated. The downside is that the signal is smoothed, reducing the computed amplitude. Furthermore, the Fixed TW often encompasses two TEPs (typically the early TEPs) in one single TW, which is intended to be exclusive to one TEP. Overall, this results in a suboptimal selection of milliseconds for evaluating the signal (the TW used to assess each TEP), leading to a loss of temporal resolution of individual TEPs.

The spatial distribution of the TEPs is also affected by the TW used, as shown in Fig. 3. The N45 potential, along with P180, exhibits broader differences among TWs due to its short duration. Despite its short duration, the spatial distribution of N45 shows some similarity between GMA and Personalized TW, as the latency of N45 in GMA is close to the median population. Additionally, the width is smaller in the GMA TW compared to the Fixed TW. The same pattern is observed with P180 and P60 (median latency close to the GMA TW latency). However, the scalp distribution of the Personalized and the GMA TWs is more distinct in the P180. This wave is a relative slow potential, so if the TW is not precisely centered on the peak and the width of the TW is small, there will be more differences between TWs. A similar situation rises with the P140 potential. Lastly, for the N100 wave, statistically significant differences are only found when comparing the Fixed TW with the other two (GMA and Personalized TW). No differences are found between GMA and Personalized TW because the latency of N100 has little variability, and the latency of GMA TW is again similar to the median latency of the subjects. Therefore, both features (similar latencies and small variability across subjects) make the GMA and Personalized TW appear alike and highly overlapped.

### Signal characterization

When examining the statistical topography and the topographical maps illustrating the differences between SCZ and HC populations for the three different TWs (see Figs. 4, 5 and 6), it is evident that the differences across the cerebral cortex between the two populations are maximized when a personalized TW is employed.

While the most pronounced differences arise with a Personalized TW, consistent trends in the same regions are evident across all three TWs. Indeed, the topographical localization of the TEPs is similar among TW methods. It indicates that all methodologies are efficient in the localization of the TEPs, but a Personalized TW provides more accurate topographical localization of the TEPs. Moreover, as stated before, there is variability in the latency of the TEP. Due to this variability, the localization, and consequently; the characterization of the TEP, is more precise when using a Personalized TW rather than a TW with common latencies for all subjects.

With a Fixed TW, only a few differences are observed in N100 and P180 potentials, whereas the differences in both TEPs are more pronounced for the GMA. Both GMA and Personalized TWs also enable the analysis of the transition to N100 and P180, incorporating a novel TEP: the P140 [33, 34]. Furthermore, differences in the N45 wave between populations are observed in the two data-dependent TWs (GMA and Personalized TW).

Less activity is observed for the SCZ group in the N45 at the right-frontal electrodes. Similarly, Noda et al., 2021 [23] found similar decrease, although in different region (left DLPFC), which is thought to be associated with GABA A-receptor mediated inhibition [17]. Regarding the N100, greater absolute amplitude values are obtained in frontal and occipital regions for SCZ, also reported by Dhami et al., 2020 [20] and Voineskos et al., 2019 [19] when patients with major depressive disorder (MDD) disorder were evaluated. MDD, a psychotic disorder, is believed to share similar neurophysiological features with SCZ [8]. N100 amplitude following single TMS pulses was directly related to the glutamate levels and inversely related to GABA levels in the medial prefrontal cortex, indicating that larger N100 TEP amplitude reflects greater cortical excitability [16]. Similarly, benzodiazepines are associated with reduced TEP amplitudes [35, 36]. Therefore, the larger N100 in patients may reflect a hyperactive, hyperexcitable cortical state, possibly related to a Glutamate/GABA imbalance. The relationship between N100 and depression was also evaluated in an MDD population before and after treatment, revealing greater (more negative) N100 amplitudes before treatment. This suggests a cortical excitation and inhibition imbalance during more severe depression [37]. Concerning the P140 and P180 potentials, the SCZ group show greater values at the central electrodes, which has been suggested to be linked with a late excitatory potential or cortical disinhibition [23]. It should be noted that, although reduced event-related potential amplitudes during cognitive tasks, such as P300 paradigms, are a well-replicated finding in schizophrenia, TMS-induced potentials do not involve a cognitive component but directly reflect cortical excitability. A hyper-active basal cortical state, as suggested by the decreased GABA activity reported in schizophrenia [38], would lead to increased TEP amplitudes and, as a consequence of a lesser margin for cognitive activation, reduced amplitudes of event-related potentials during cognitive tasks.

In relation to the TEP amplitude (Fig. 7), larger values are achieved when using the Personalized TW. Moreover, the values also increase with the GMA TW when compared to the Fixed TW. This is attributed to the fact that the TEPs are more precisely captured with an individualized TW, and the narrower width, allows for exclusive consideration of the TEP within the TW; as evidenced by the statistical significance between Fixed and Personalized TW. In addition, when examining P140 wave at the GMA TW, it is apparent that negatives values are obtained for the SCZ population, despite the adaptation of the TEP characterization the data to some extent. This underscores the necessity of a high degree of individualization in the time windows, a point confirmed when employing the Personalized TWs.

Concerning the latencies of the TEPs in the context of the Personalized TW, it is observed that the early TEPs appear later, with statistical significance, in the HC population compared to the SCZ group. This observation may be attributed to the baseline hyperactive state of the schizophrenic brain, suggesting that when the TMS-pulse is triggered, it is transmitted faster than in the HC population [39, 40]. We can only speculate that short-latency potentials would appear faster due to a locally more pronounced inhibitory deficit: the TMS pulse is applied on the prefrontal cortex, where GABA deficits have been consistently reported [10]. Conversely, latency of later potentials may be less affected, possibly due to a smaller inhibitory deficit in neighboring regions where the TMS pulse would spread.

### Limitations and future research

It is important to consider the limitations of this study when interpreting the results. First, the small sample size may limit the detection of statistically significant differences across populations and time windows. This limitation should be taken into account when discussing the results. Increasing the sample size could enhance the statistical power and robustness of the findings. Second, the absence of neuronavigation for localizing the left DLPFC might introduce variability in the stimulation site. However, our approach aligns with previous studies in the field, where the coil placement between the F3 and F5 electrodes is considered to provide the most accurate estimation of the left DLPFC [24, 25]. Third, despite efforts to minimize TMS-induced somatosensory and auditory artifacts in the TMS-EEG signal, complete elimination cannot be guaranteed. Fourth, our sample consists of medicated individuals, making it challenging to disentangle the effects of medication from the observed differences. A treatment-naïve sample could provide additional insights into the impact of medication. Lastly, while our pre-processing follows the approach of Rogasch et al., 2014 [28], different pre-processing approaches exist in the literature [41] and future studies could explore the effects of different pre-processing methods on TEPs characterization.

Furthermore, future research employing a larger sample size and considering the treatment status of the schizophrenic population could yield additional measurements to further validate the identified differences.

## Data Availability

The data that support the findings of this study are available from the corresponding author upon reasonable request.
